# The Accuracy of Transrectal Ultrasound-Guided Biopsy for Decision Making in Prostate Cancer

**DOI:** 10.5812/numonthly.19476

**Published:** 2014-07-05

**Authors:** Ali Reza Ghadian, Fatemeh Heidari, Ali Reza Afkhami

**Affiliations:** 1 Nephrology and Urology Research Center, Baqiyatallah University of Medical Sciences, Tehran, IR Iran

**Keywords:** Neoplasms, Prostate, Cancer

## Abstract

**Background:**

The most important surgical complications of renal transplantation are stenosis and obstruction of anastomosis of the ureter to the bladder. Hence, routine use of ureteral stents to prevent such complications seems logical; however, the optimal time to remove the ureteral stent is still controversial.

**Objectives:**

The purpose of this study was to compare the benefits and complications of the early or delayed ureteral stent removal post-transplantation.

**Patients and Methods:**

All patients who underwent kidney transplantation in Modarres Hospital from May 2011 through March 2012 were recruited. The patients were allocated to three groups. Ureteral stent removed 10, 20, and 30 days after transplantation in groups one, two, and three, respectively.

**Results:**

A total of 91 patients had undergone renal transplantation in our center. Ureteral stent was removed at 10, 20, and 30 days after surgery. Urologic complications among the three groups included hydronephrosis, urinoma, and collection around the graft; there was no statistically significant difference among study groups with regard to frequency of complications.

**Conclusions:**

We can remove the ureteral stent at shorter interval after renal transplantation with no increased risk of urologic complications.

## 1. Background

In the recent decades, renal transplantation surgery has increased dramatically (1). On the other hand, urologic complications following surgery are inevitable (1, 2). These complications can include urinary leakage from the ureteral anastomosis, fistula, stenosis, and obstruction of the urinary tract, especially at ureteral anastomosis (1-3).

The most important surgical complications are stenosis and obstruction of anastomosis of the ureter to the bladder that can occur in 2% to 7.5% of cases (1, 4). These complications cause high morbidity, increased hospitalization time, and subsequent increase in cost (1, 2). Therefore, routine use of ureteral stents during kidney transplantation as prophylaxis to prevent such complications seems logical (1-3).

Ureteral stent placement to reduce surgical complications does not cause significant increases in costs (5); moreover, postoperative antibiotic prophylaxis decrease urinary tract infection (UTI) rate after ureteral stent placement (5, 6). Ureteral stent removal is an endoscopic procedure; thus, ureteral stenting to avoid ureteral anastomosis complications is a cost-effective procedure (1, 2, 5); however, use of ureteral stents is associated with some complications (1, 3, 5, 6). The major complication during ureteral stenting is increased rate of UTI (5, 6). Other complications include stent migration, persistent hematuria, bladder irritation caused by stent, and the complications during stent removal (5, 6).

Ureteral stent is usually removed four to six weeks posttransplantation (7); however, the optimal time of ureteral stent removal is still controversial and has not been specified yet (1, 7).

## 2. Objectives

The purpose of this study was to compare the benefits and complications of the early or delayed ureteral stent removal posttransplantation as early ureteral stent removal decreases some complications such as UTI, bladder irritation symptoms, and persistent hematuria. Early ureteral stent removal reduces the risk of stent crusting and makes stent removal easier. If ureteral stent is removed in the early period after surgery, hospitalization time and need for readmission for stent removal would be reduced.

## 3. Patients and Methods

All the patients who underwent a kidney transplantation from May 2011 to March 2012 in Modarres Hospital, Tehran, Iran, were recruited. Exclusion criteria were previous history of kidney transplant rejection, serum creatinine levels higher than 3 mg/dL before ureteral stent removal, significant hydronephrosis, urinoma, or substantial collection around the graft in the ultrasonography (US) before stent removal, previous history of chemotherapy or radiotherapy to the pelvis, and receiving kidney from cadaver.

Before ureteral stent removal, serum creatinine levels, urine culture, and renal US to assess of hydronephrosis, urinoma, and collection around the graft were performed. Then the patients were randomly allocated to three groups according to random numbers table. The ureteral stent was removed 10, 20, and 30 days after transplantation in groups 1, 2, and 3, respectively. The patients were reassessed one month after ureteral stent removal. The data were analyzed by SPSS (version 18. SPSS Inc., Chicago, IL, USA).

## 4. Results

Based on eligibility criteria, the total number of patients in this study was 91 among which 54 were males and 37 were females. The patients’ sex in each group is shown in (Table 1). Patients mean age was 41.1 ± 14.2 years in group 1, 38.03 ± 13.49 years in group 2, and 43.7 ± 14.37 years in the group 3. Minimum and maximum age of the patients in all three groups are shown separately in Table 2.

UTI was confirmed by urine cultures in six patients in group 1, three patients in group 2, and eight patients in group 3 (a total of 17 patients) before stent removal. After ureteral stent removal, UTI was diagnoses by urine culture in three patients in group 1 and two patients in group 2 (a total of five patients) (Table 3). According to the US findings before ureteral stent removal, one patient in group 1 and one in group 3 had mild hydronephrosis (a total of two patients), one in group 1, one in group 2, and two in group 3 had urinoma (a total of four patients), and one patient in group 1 had a collection around the graft.

One month after ureteral stent removal, US showed mild hydronephrosis in two patients in group 1, one patient in group 2, and two patients in group 3 (a total of six patients), uremia in one patient in group 2 and two patients in group 3 (a total of three patients), and collection around the kidney in one patient in group 2. 

Mean creatinine in patients before removal of the stent was 1.3 mg/dL (range, 1-1.8) in group 1, 1.44 mg/dL (range, 1.1-2.8) in group 2, and 1.37 mg/dL (range, 1-2.4) in group 3. The patients' mean creatinine after stent removal was 1.37 mg/dL (range, 1-1.9) in group 1, 1.42 mg/dL (range, 1-2.5) in group 2, and 1.45 mg/dL (range, 1-2.2) in group 3.

The mean age had no statistically significant differences among the three groups (P value = 0.25). Presence of active UTI before stent removal had no statistically significant differences among the three groups (P value = 0.22).

With regard to hydronephrosis, collection around the kidney, and urinoma, no statistically significant difference was detected among study groups before ureteral stent removal (P value of 0.59, 0.35, and 0.76, respectively).

After stent removal, there was no significant difference among three groups with regard to hydronephrosis, collection around the kidney, urinoma, and UTI (P value of 0.71, 0.37, 0.76, and 0.22, respectively). The abovementioned results are summarized in Tables 4 and 5.

Regarding creatinine, there was no statistically significant differences among the three groups before and one month after stent removal (P value of 0.15 and 0.42, respectively). The result are shown in Table 6.

**Table 1.** Distribution of Patients in Study Groups With Regard to the Sex^a^

Groups	Female	Male
**Group 1**	12 (40)	16 (60)
**Group 2**	14 (45.2)	17 (54.7)
**Group 3**	11 (36.7)	19 (63.3)

^a^Data are presented as No. (%).

**Table 2.** Distribution of Patients in Study Groups With Regard to the Age

Groups	Min	Max	Mean ^a^
**Group 1**	20	68	41.1 ± 14.2
**Group 2**	15	68	38.03 ± 13.49
**Group 3**	15	64	43.7 ± 14.37

^a^ Data are presented as mean ± SD.

**Table 3.** Frequency of Positive Results in Urine Culture Among Study Groups^a^

Groups	Before Remove of Stent	After Remove of Stent
**Group 1**	6 (20)	3 (10)
**Group 2**	3 (9.7)	2 (6.5)
**Group 3**	8 (26.7)	-
**Total**	17 (18.6)	5 (5.4)

^a^Data are presented as No. (%).

**Table 4.** Patients Features Before Stent Removal ^a^

Before Stent Removal	Group 1, n = 30	Group 2, n = 31	Group 3, n = 30	P value
**Urine Culture, positive results**	6 (20)	3 (9.7)	8 (26.7)	0.22
**Hydronephrosis**	1 (3.3)	0	1 (3.3)	2.59
**Urinoma**	1 (3.3)	1 (3.2)	2 (6.7)	2.76
**Collection**	1 (3.3)	0	0	2.35

^a^ Data are presented as No. (%).

**Table 5.** Patients Features After Stent Removal^a^

After Stent Removal	Group 1, n = 30	Group 2, n = 31	Group 3, n = 30	P value
**Urine Culture, positive results**	3 (10)	2 (6.5)	0	0.22
**Hydronephrosis**	2 (6.7)	4 (12.9)	3 (10)	0.71
**Urinoma**	1 (3.3)	1 (3.2)	2 (6.7)	0.76
**Collection**	0	1 (3.2)	0	0.76

^a^ Data are presented as No. (%).

**Table 6.** Amount of Creatinine Before and After Stent Removal

Creatinine	Group 1, n = 30	Group 2, n = 31	Group 3, n = 30	P value
**Before Stent Removal**	1.30 ± 0.20	1.44 ± 0.32	1.37 ± 0.26	0.15
**After Stent Removal**	1.37 ± 0.19	1.42 ± 0.31	1.45 ± 0.23	0.42

## 5. Discussion

Urologic complications following renal transplantation surgery can cause considerable morbidities (1-6). Two major complications are stenosis of the ureter to urinary bladder anastomosis and urinary leakage (3). The use of ureteral stents during the transplantation to prevent complications has been confirmed in many studies (1, 3, 5); however, there is no consensus about the ideal time of the ureteral stent removal (3). According to some studies, stent is usually removed four to six weeks after surgery (7). Typically, the stent is removed by endoscopic procedures (2, 5); however, in some studies, external stenting procedures were evaluated to remove the stent when the patient could not undergo cystoscopy. In a study conducted by Minnee et al. in Amsterdam, external stent removal after five days was associated with satisfactory results (3). In a study conducted in 1995, Bassiri et al. studied the ureteral stent remaining for six to eight weeks (8). When remained for a longer period, stents were associated with some complications such as increased risk of UTI, hematuria, bladder irritation symptoms, and complications due to stent crusting (1, 3, 5, 6). For example, the rate of UTI in patients with ureteral stents in studies by Bassiri et al. and Vrema et al. was 33% and 25%, respectively (8, 9). On the other hand, there are studies such as the one by Pleass et al. that did not report an increased risk of UTI in patients with ureteral stent (10). In our study, the rate of UTI after ureteral stent removal showed no difference among study groups; however, the rate of UTI had decreased from 18.6% before stent removal to 5.45% at one month after stent removal. In Vrema et al. study, patients were allocated to two groups and the ureteral stents were removed two and four weeks after surgery (9). In their study, the rate of UTI was higher in those their stent was removed after four weeks of surgery; however, it was not statistically significant. They removed stents at the time of discharge from the hospital, which was cost-effective. They stated that ureteral stent removal within two weeks of surgery does not increase the risk of UTI (9).

In our study, ureteral stents were removed at 10, 20, and 30 days after surgery. Creatinine before and one month after stent removal had no statistically significant differences among the study groups. Urologic complications among the three groups were hydronephrosis, urinoma, and collection around the kidney that had no statistically significant difference among the study groups. In patients that their stent was removed ten days after transplantation, the stent was removed at the time of discharge from the hospital and as a sequence, costs were reduced.

It can be concluded that according to the routine placement of ureteral stents during kidney transplantation surgery, we can remove the ureteral stent after surgery at shorter interval without any increased risk of urologic complications.
